# Measuring creative imagery abilities

**DOI:** 10.3389/fpsyg.2015.01591

**Published:** 2015-10-21

**Authors:** Dorota M. Jankowska, Maciej Karwowski

**Affiliations:** Creative Education Lab, Department of Educational Sciences, The Maria Grzegorzewska UniversityWarsaw, Poland

**Keywords:** creative imagination, vividness, originality, transformativeness, TCIA

## Abstract

Over the decades, creativity and imagination research developed in parallel, but they surprisingly rarely intersected. This paper introduces a new theoretical model of creative visual imagination, which bridges creativity and imagination research, as well as presents a new psychometric instrument, called the Test of Creative Imagery Abilities (TCIA), developed to measure creative imagery abilities understood in accordance with this model. Creative imagination is understood as constituted by three interrelated components: vividness (the ability to create images characterized by a high level of complexity and detail), originality (the ability to produce unique imagery), and transformativeness (the ability to control imagery). TCIA enables valid and reliable measurement of these three groups of abilities, yielding the general score of imagery abilities and at the same time making profile analysis possible. We present the results of nine studies on a total sample of more than 1700 participants, showing the factor structure of TCIA using confirmatory factor analysis, as well as provide data confirming this instrument's validity and reliability. The availability of TCIA for interested researchers may result in new insights and possibilities of integrating the fields of creativity and imagination science.

## Introduction

**Figure 1:**
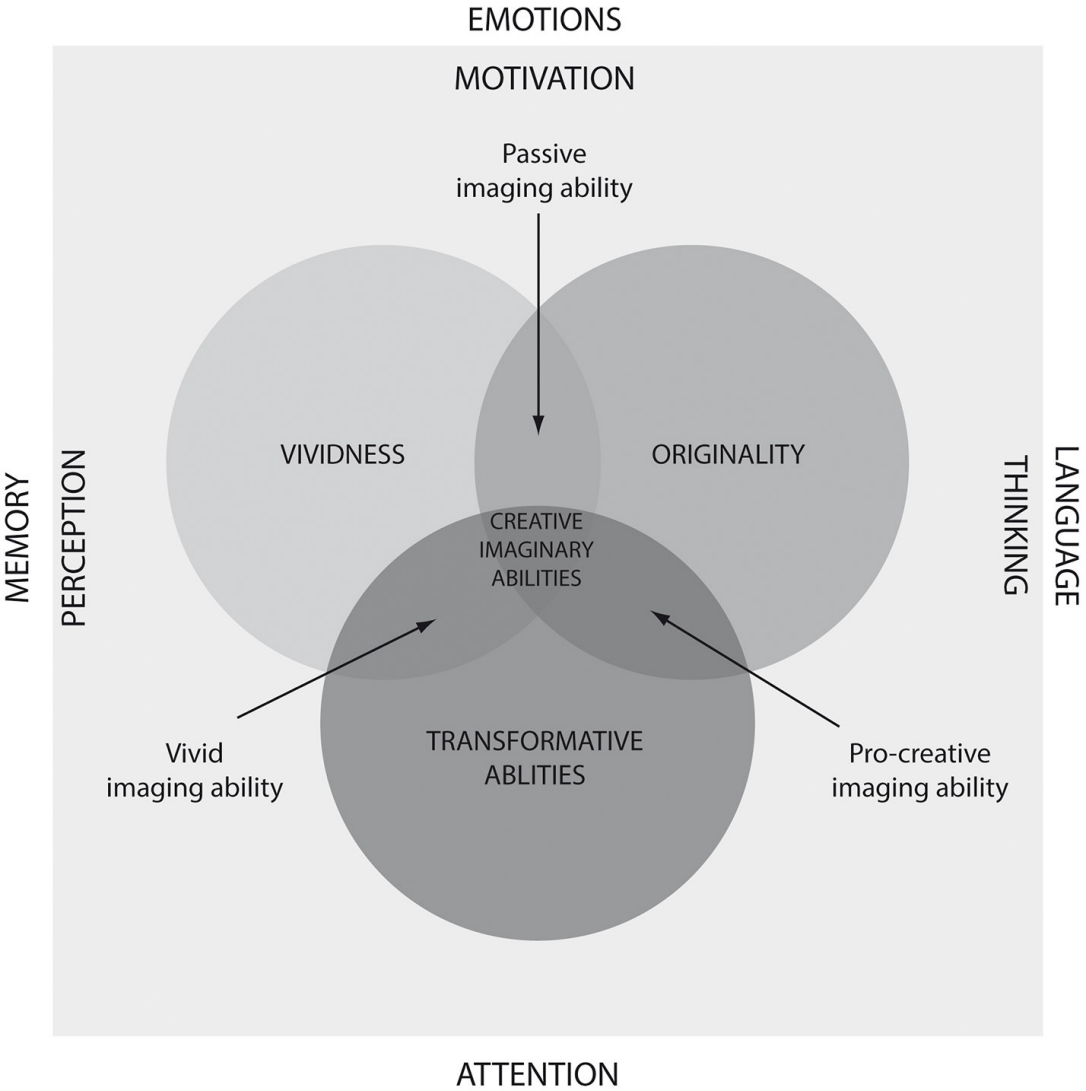
**The conjunctional model of creative imaging ability**.

Imagination pervades human experience. The activity of visual imagination encompasses creating, interpreting, and transforming vivid mental representations (Thompson et al., 2011). Its creative function, which stems from engagement in the creative process, is most often discussed in connection with the imaginary games of childhood (Singer and Singer, 1992; Hoff, 2005) as well as artistic and scientific work (Rothenberg, 1995; Root-Bernstein, 2014). However, the belief that creative imagination is one of the major human abilities contributing to the effective use of the creative potential (Runco et al., 1998) is not a matter of recent years only. The first documented study on imagination was conducted among scientists nearly one and a half centuries ago (Galton, 1880), and with the development of research on creativity test instruments measuring visual creative imagination were created. However, the existing tests do not take into account the complexity of creative imagination, which became an impulse for developing the Test of Creative Imagery Abilities (TCIA), whose theoretical assumptions as well as selected aspects of validity and reliability we present in this paper. The instrument we propose enables profile analysis of visual creative imagination, thereby treating imagination as a complex and multidimensional disposition comprising specific characteristics (vividness, originality, transformative ability) distinguished in the conjunctional model of creative imaging ability. In this model, creative imagination is defined as ability to create and transform representations that are based on the material of past observations but that significantly transcend them—by creating the so-called creative representations (see Dziedziewicz and Karwowski, 2015; Figure 1). Although creative imagination understood in this way is part of the broad construct of creative cognition (Finke et al., 1992), we perceive creative imagination in a more narrow way, than we do creative cognition.

### Problems with measures of creative imagination

Test-based research on creativity originated with Guilford's (1950) theory of divergent thinking. With time, Guilford's tasks measuring the characteristics of divergent thinking gave rise to numerous tests, such as the Torrance Tests of Creative Thinking (TTCT; Torrance, 1974) or Thinking Creatively in Action and Movement (TCAM; Torrance, 1981). For many years, this tradition of creativity research remained the dominant approach. And even though imagination measurement in psychology and related sciences has a longer tradition than research on divergent thinking (Galton, 1880), it was the post-Guilfordian orientation that exerted considerable influence on the testing of creative imagination, not the other way around. The influence was so strong that the contribution of creative imagination was included in the first tests for the assessment of divergent thinking, an example being the “Imaginative Stories Task” in the Minnesota Test of Creative Thinking (MCTC; Torrance, 1962; Goldman, 1965; Millar, 2002), the original version of TCAM. The combination of these abilities in divergent thinking resulted in a blurring of the concept of imagination, previously well defined in the literature. Interestingly, many questionnaires for exploring visual imagination were developed in parallel (e.g., Sheehan, 1967; Marks, 1973; Heckler et al., 1993), measuring mainly the following: (1) imagery vividness—the clarity, complexity, and elaboration of the imagery generated; (2) imagery control—the ability to manipulate the imagery generated; and (3) imagery style—a preference for imagery-based or verbal strategies of encoding and processing information (MacInnis, 1987). The assessment criteria in the newly developed test measures were nearly identical with those in typical divergent thinking tests, for example: flexibility, elaboration, originality, asymmetry, and abstraction in the Franck Drawing Completion Test (FDCT; Schaefer, 1970; Anastasi and Schaefer, 1971), flexibility, elaboration, and originality in the Visual Imagination Test (VIT; McHenry and Shouksmith, 1970), or flexibility and originality in the Creative Imagination Test (CIT; Schubert, 1973). On the other hand, the influence of Guilfordian tests on the practice of testing and creative imagination assessment may not be so obvious as it is described to be. Long before Guilford's (1950) famous address, which gave impulse to the development of the psychology of creativity, Simpson (1922) presented the Test for Creative Imagination (Visual), in which the counterpart of transformativeness was the *creative changes* indicator, which was the prototype for the flexibility of thinking. This measure was computed based on the product of the number of all the drawings produced in the test and the number of changes between the drawings (i.e., the number of transition moments between different categories). It can therefore be supposed that first definitions of imagery transformation ability were positioned within the area of meanings and their interpretations, just like the flexibility of thinking.

With time, many empirical studies appeared that demonstrated a weak relationship between imagination and divergent thinking (Parrott and Strongman, 1985; Campos and Perez, 1989; Campos and González, 1993), which is confirmed by the meta-analysis summing up these studies (LeBoutillier and Marks, 2003). It therefore became justified to treat these constructs as distinct and relatively independent components of creativity, each having its own measurement specificity. Nevertheless, the influence of the post-Guilfordian tradition was still so strong that even after the publication of the Test of Creative Thinking by Jellen and Urban (TCT-DP; Jellen and Urban, 1986), which, in some sense, overcame the dominance of the Guilfordian approach in thinking about creativity, the scoring criteria in new creative imagination tests were still a reproduction of fluency, flexibility, originality, and elaboration. For instance, in Prueba de Imaginación Creativa (PIC; Artola et al., 2004) five scales were distinguished, of which four are repetitions of the components of divergent thinking: fluency of ideas, flexibility of thinking, originality of the responses, elaboration of the responses, and use of creative details (color, shadows, expansiveness, rotations, new perspectives). And while references to fluency, which can be linked with the generativity (fertility) of imagination, are to some extent justifiable, defining the originality of the generated imagery in terms of the rarity of their occurrence is an oversimplification that results from copying the scoring criteria for divergent thinking. The creative aspect of imagery manifests itself in generating new ideas and hypotheses, which are rare by nature, but above all they are innovative (Ward, 1994; Magid et al., 2015). This way of thinking about the originality of imagery is visible in the Test of Creative Imagination (TCI; Karwowski, 2008a,b), where the participant's task is to imagine and draw schematic drawings representing something that does not exist but, in the participant's opinion, should exist.

Reproducing the scoring criteria for divergent in creative imagination tests resulted in the similarity of test tasks. For example, the FDCT matrix is almost an exact copy of the matrix in the figural part of TTCT—Picture Completion. The situation is similar in the case of PIC and the Test of Creative Imagination (TCI, Ren et al., 2012). They all consist of incomplete figures to be completed and captioned, the difference being that FDCT has 12 figures, PIC has 4, and in TTCT and TCI there are 10 of them. This is undoubtedly a reference to the Sketches Test, in which the participant is given a simple basic figure, such as a circle, that he or she is supposed to complement in such a way as to produce a recognizable sign (Guilford and Hoepfner, 1966). A similarity is also observable in verbal tasks. In the version of PIC that is intended for children, the tasks in the verbal part require describing: (1) the possible consequences of all squirrels turning into dinosaurs, (2) new applications of plastic pipes, and (3) various endings of a situation presented in a picture. In the verbal part of the TCI, participants generate alternative endings for a briefly outlined story. It is not difficult to notice that these are typical tasks from the Remote Consequences Tests of the Unusual Uses Tests (Guilford, 1967). However, they are not always a copy of Guilford's tasks. In the TCI test sheet there are 16 elements—in groups of four: dots, semicircles, straight lines, and curved lines—out of which it is easy to make schematic drawings. Just like in the Make a Figure Test, simple linear elements are provided; however, the essence of the task is not to contrive to arrange as many complex figures as possible out of those elements (Guilford, 1967) but to use them for schematically presenting a generated mental image. This shows that the problem of creative imagination tests does not lie in their being inspired by tasks invented by Guilford but in the frequently rather mechanical imitation of their specificity and scoring.

Another problem connected both with the specificity of tasks and with their scoring, is the construct validity of creative imagination tests. Some of those instruments have unclear theoretical roots. FDCT originally served to carry out projective studies of masculinity and femininity characteristics (Franck and Rosen, 1949; Harkey, 1982). Barron (1958) proposed a new version of the test; drawing on the Guilfordian definition of originality, he developed the Originality Scale of FDCT, which placed emphasis on the originality, complexity, and asymmetry of the drawings made. The use of Guilford's theory once again confirms the strong domination of this orientation in the psychology of creativity, since at least two comprehensive theories of creative imagination were already in existence at that time—Ribot's (1906) and Vygotsky's (1930/2004, 1931/1991).

Another problem of creative imagination tests is the time limitations on administering them—from 10 min in PIC, modeled on TTCT, to 30 min in the TCI. Thus, they are mostly tests of speed (MCTC; FDCT; PIC; TCI). As a result, solving these tests requires, above all, quick reaction to tasks. The result obtained in a test may therefore depend not on the actual level of imagery abilities but on intellectual mobility. Individuals with a higher speed of intellectual work will do more test tasks in a specified unit of time, which again indirectly relates to the fluency of thinking, making these tests closer to classic Guilfordian tests in terms of scoring.

The next charge—serious but overlooked by many researchers—is associated with imagery transformation abilities; it concerns the aprocessual character of creative imagination: that is, making inferences about the transformations performed exclusively on the basis of their final outcome, being a reflection of the imagery generated. The simplest schema of inference about transformations is an analysis of the transition from the original image to its final form. In figural tests based on the Sketches Test (FDCT, PIC, TCI), inference about transformations is based on the analysis of changes in the stimuli evoking the imagery; for example, in FDCT the participant gets one point on a three-point scale for making a drawing that is elaborate in form and not rigidly based on the initial symbol. This is a risky kind of inference about imagery transformation, since it concerns the elaboration and complexity of an image—which determine the imagery vividness index—to a greater degree than the transformation abilities responsible for the result of the process of reconfiguring or recombining concepts (Ward, 1994). It is therefore legitimate to venture the statement that a majority of creative imagination tests place emphasis on measuring he ability to generate vivid and complex imagery as well as its originality.

The problems described, associated with the measurement of creative imagery abilities, were an impulse for us to develop a new instrument. Drawing on the long tradition of research on visual and creative imagination and at the same trying to avoid the shortcomings of the existing tools described above, we developed the TCIA, whose assumptions and selected aspects of validity and reliability we will present in the further sections of this paper.

### Assessment of visual creative imagination—a new measurement instrument

The TCIA measures the intensity of three characteristics of creative imagination distinguished in the conjunctional model of creative imaging ability: (1) vividness—the ability of generating clear and distinctive imagery characterized by high complexity, specificity, and elaboration; (2) originality—the ability of generating creative imagery characterized by novelty; and (3) transformative ability—the ability of modifying and transforming the imagery generated (Dziedziewicz and Karwowski, 2015, see also Figure 1). The test can be used in individual and group studies at different age levels—from about the age of 4 years to late adulthood.

The TCIA test booklet is in A3 format and consists of seven tasks. The first stage of solving each task has an exploratory character. The participant (in a group study) is supposed to give, in an oral or written form, as many images generated on the basis of a simple graphic sign, called the initial figure. Next, he or she selects the most original of the images given and, on its basis, makes a drawing accompanied by a brief description. The instruction stresses the possibility of elaborating and changing the selected image and adding any elements to it in such a way as to create something even more original: “You will find an unfinished drawing on every page of the test. Please write what it reminds you of. The more unusual ideas, the better. Next, underline the idea that you like most. Think of what you can change in it, reshape, and develop it in order to create something even more unique. Draw in the box and give your drawing a title. Good luck!” (see Figure 3). In an individual interview, the researcher writes down the participant's answers on a specially prepared answer sheet. Regardless of the manner of testing, the time allowed for solving the test is not limited. Usually, solving the TCIA does not require more than 20 min.

**Figure 2:**
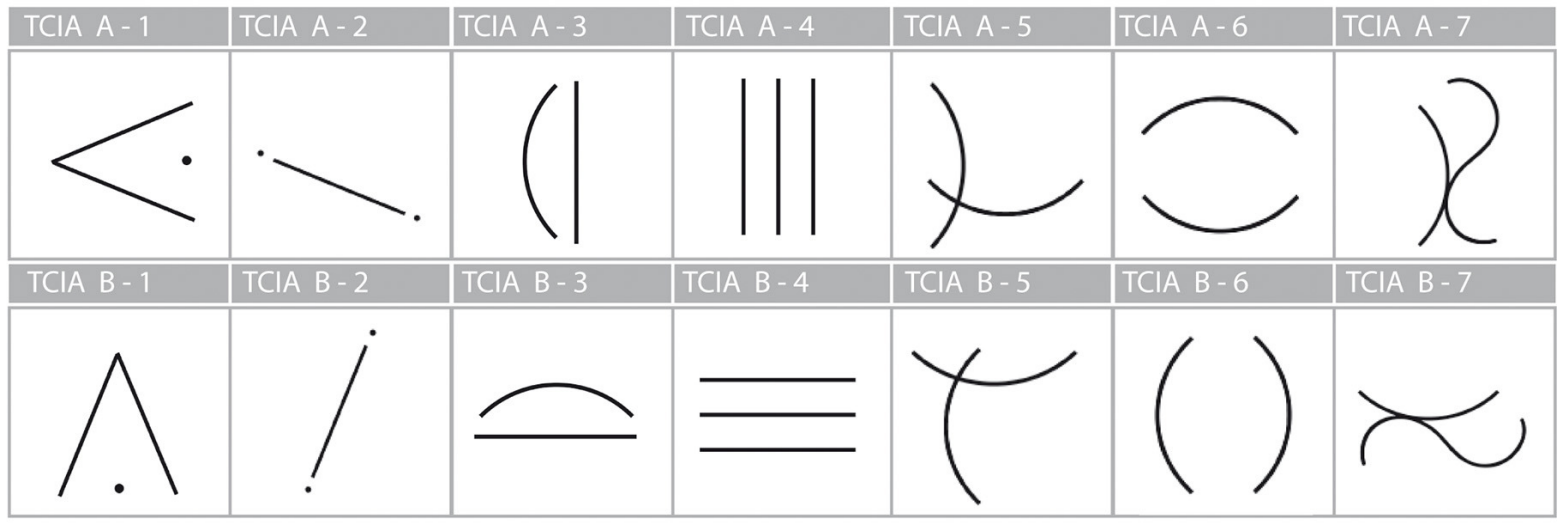
**The TCIA test booklet**.

The test has two parallel versions (A and B) that differ only in the position of the signs—in version B, each initial imagery-evoking sign is rotated by 180 degrees (see Figure 2).

**Figure 3:**
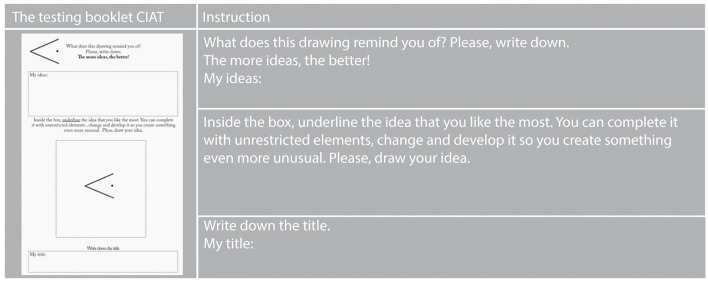
**Initial signs of TCIA**.

Most tests measuring creative imagination do not have alternative versions (e.g., FDCT, TCI), which was the main impulse to start work on developing parallel versions of TCIA. The possibility of using the parallel versions of the test is of great importance in educational assessment, particularly when checking the effectiveness of various interventions. Their use in experiments involving the initial and final measurements of the dependent variable eliminates the necessity of applying the same instrument and thereby increases the validity of the design.

The drawings and descriptions of imagery made in TCIA are assessed on three scales based on the conjunctional model of creative imaging ability (the Vividness scale; the Originality scale; the Transformativeness scale). Each scale is scored according to the criteria discussed in detail and illustrated with examples in the test manual (Jankowska and Karwowski, 2015). According to these criteria, it is possible to score 0, 1, or 2 points on each scale for a single drawing. The scores on scales are computed by adding up the points given to all the drawings. The total score is the sum of points obtained on the scales: Vividness, Originality, and Transformative Ability. Additionally, the analysis may also cover the index of imagination generativity—Imaginative Fluency (see Table 1).

**Table 1:** **Example TCIA assessment criteria**.

**Scoring**	**Vividness**	**Originality**	**Transformativeness**
0	The original figure has not been supplemented, but was interpreted, i.e., it was given the title	Presentation of common objects (things, plants, animals, people, places). Their shapes, functions, and properties are real, and their activities, processes, states, and events are typical	Multiplication of the original figure
1	Simple, frequently schematic completion of the original figure	Individual, simple modifications of shape, functions, and properties of widely known objects (things, plants, animals, people, places) as well as typical activities, processes, states, and events;	Recreation, simple completion of the original figure, and adding to it a relatively independent object(s)
2	Complex, rich in detail completion of the original figure	Complex, significantly altered with respect to reality, modification of shape, functions, and properties of widely known objects (things, plants, animals, people, places) as well as typical activities, processes, states, and events	Complex modification of the original figure—its multi-aspect elaboration

**Figure 4:**
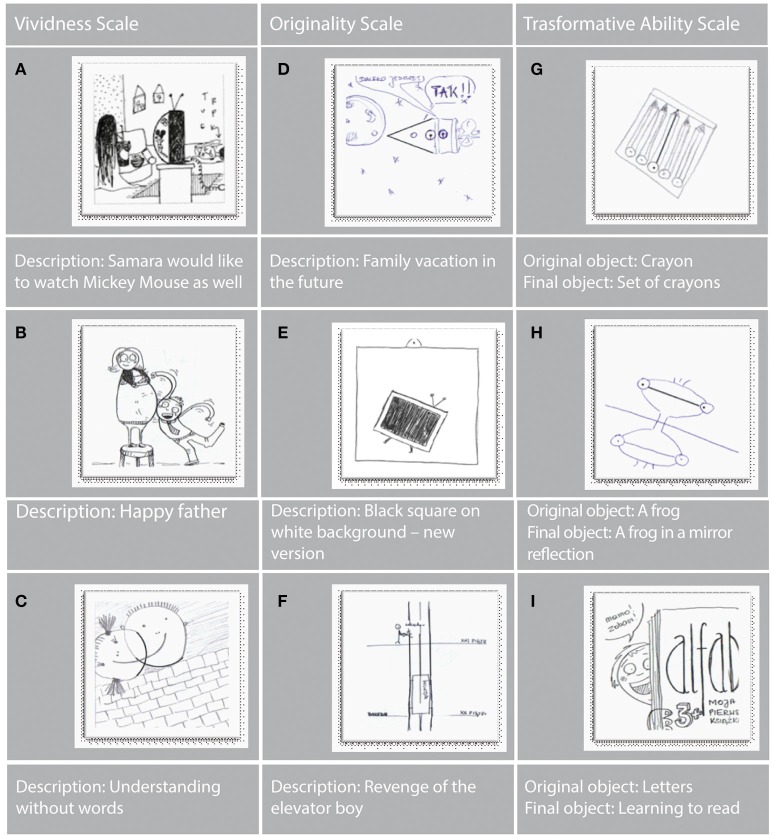
**Example drawings from TCIA**. See text for A-I description.

The Vividness scale measures the degree of visualization and elaboration of the imagery generated. A high level of vividness is recognized, for instance, by the following: (A) an abundance of detail in the completion of the initial figure; (B) a clear depiction of motion and dynamics in the drawing; and (C) a complex presentation of metaphorical and symbolic content. The Originality scale measures the novelty of the imagery generated. A high level of originality is attested, for example, by: (D) the depiction of new objects, activities, processes, and events in the drawing that differ considerably from the actually existing ones; (E) surprising and novel presentation of cultural artifacts such as works of art; (F) amusing presentation of contents, suggesting a good sense of humor. The Transformativeness scale measures the ability of modifying the imagery generated. The scoring criteria refer to basic operations of transforming visual imagery, such as: (G) multiplication—multiplying an element of the image; (H) hyperbolization—excessive distortion of proportions, for example by emphasizing an element of the image; (I) amplification—adding detail to the image (see Figure 4).

In order to establish the structure of imagery abilities characteristic for a particular person, TCIA scores can be subjected to profile analysis. Each imagery ability is then assessed against the backdrop of the person's other imagination-related skills or against the norms determined for a certain population. The profile thus obtained is useful in predicting the further development of imagination and in deciding on the direction of supportive and stimulatory interventions.

In profile-based analysis, high scores on all the three scales attest creative imagery abilities. In the case of vivid imaging ability, the imagery generated is expressive but imitative—it is almost an exact reflection of previously perceived and memorized images. In cases of this kind, people should be inspired to creatively combine, non-typically link, and modify the generated images so as to give them features of novelty. Individuals with pro-creative imaging ability should be encouraged to create expressive imagery, add detail to it, and make it dynamic. By contrast, in the case of passive imaging ability profile, stimulatory interventions should focus on developing the ability of transforming imagery in unconstrained and miscellaneous ways.

## The present studies

The research program presented below was aimed at testing the psychometric properties of the new test. In nine studies, on a total sample of 1700 participants, we tested criterion validity, juxtaposing TCIA results with other measures of imagination and creative abilities (Studies 1–5) and the discriminant validity of TCIA (Study 6), checking whether and to what extent TCIA dimensions are related to intelligence and school achievement measured using standardized tests as well as GPA. In the next step, using aggregated data, we tested the construct validity of the new test by performing confirmatory factor analysis. We also show the measurement invariance of TCIA among women and men as well as the relations between age and creative imagination.

The other objective of our analyses was to test the reliability of TCIA. In Study 7, we demonstrate the consistency of trained judges' evaluations on TCIA based on the manual (Jankowska and Karwowski, 2015). Study 8 is devoted to the analysis of test-retest reliability, and in Study 9 we present test-retest relations, with version B of TCIA used apart from version A. We conclude the reliability analyses by reaching for aggregated data from all the studies presented in this paper and we present the internal consistency of TCIA scales assessed using a more traditional method (Cronbach's α) as well as the more modern composite reliability (*H*; Hancock and Mueller, 2001), which is the outcome of confirmatory factor analysis. Table 2 provides an overview of all studies with descriptive statistics.

**Table 2:** **Summary of studies presented in this article, together with sample sizes, instruments, and descriptive statistics**.

**Goal**	**Study**	***N***	**Method used**	**Dimension assessed by other instruments**	**Vivid *M* (*SD*)**	**Orig. *M* (*SD*)**	**Transf. *M* (*SD*)**
Criterion validity	1	100	Vividness of Visual Imagery Questionnaire (*M* = 119.87, *SD* = 19.46)	Vividness of Visual Imagery	7.87 (2.13)	2.25 (2.02)	6.29 (3.92)
	2	57	Franck Drawing Completion Test (*M* = 9.60, *SD* = 3.48)	Creative imagination	7.20 (2.07)	1.95 (1.48)	4.38 (5.41)
			Generating Imaginary Animals (*M* = 0.85, *SD* = 2.19)	Creative cognition			
	3	261	Test of Creative Thinking-Drawing Production (*M* = 16.66, *SD* = 9.41)	Creative Thinking	6.46 (2.33)	1.80 (1.95)	3.62 (3.00)
	4	226	Verbal Alternate Uses Task, scored for: Fluency (*M* = 10.41, *SD* = 7.70), Flexibility (*M* = 6.62, *SD* = 3.70), Originality (*M* = 103.29, *SD* = 76.32)	Divergent Thinking	6.45 (2.51)	1.87 (2.03)	3.55 (3.19)
	5	741	Torrance Tests of Creative Thinking – figural test, scored for: Fluency (*M* = 8.53, *SD* = 7.76), Flexibility (*M* = 3.19, *SD* = 3.43), Originality (*M* = 43.63, *SD* = 50.16)	Divergent Thinking	6.89 (2.20)	1.75 (1.93)	5.17 (3.92)
Discriminant Validity	6	230	Raven's Progressive Matrices (*M* = 100, *SD* = 15)	Intelligence	6.22 (1.97)	1.48 (1.43)	3.22 (2.72)
			Test of School Achievement (*M* = 100, *SD* = 15)	School Achievement			
			Grade Point Average (*M* = 4.19, *SD* = 0.81)				
Interjudge Reliability	7	4 judges	Version A of TCIA	–	4 judges: 6.24 (1.76), 7.05 (2.06), 6.61 (2.17), 7.20 (2.30)	4 judges: 2.21 (1.41), 1.57 (1.54), 2.09 (1.71), 2.13 (1.69)	4 judges: 4.39 (3.21), 4.44 (3.89), 4.51 (3.24), 3.48 (2.52)
Test–retest reliability	8	86	Version A of TCIA used twice with 3 weeks interval	–	Test: 6.51 (2.18)	Test: 1.50 (1.74)	Test: 5.35 (3.53)
					Retest: 7.05 (1.99)	Retest: 1.98 (1.90)	Retest: 5.67 (3.35)
Correlation between parallel versions of TCIA	9	39	Version A and B of the TCIA used with 5 weeks interval	–	Ver. A: 7.20 (2.07)	Ver. A: 1.95 (1.48)	Ver. A: 4.38 (3.41)
					Ver. B: 7.13 (1.62)	Ver. B: 1.75 (1.30)	Ver. B: 4.08 (3.20)

### Criterion validity (studies 1–5)

#### Method

##### Participants

*Study 1*. The participants in Study 1 were 100 students (all of them female) aged 19–40 years (*M* = 22.73, *SD* = 4.71). They were students of social sciences at several universities in a big city in central Poland.

*Study 2*. The participants in Study 2 were 57 female students of education and teaching, aged 20–24 years (*M* = 20.85, *SD* = 0.59). They studied at a university of education in Warsaw, the capital of Poland.

*Study 3*. The participants in the third study were 261 children (110 girls) aged 5–7 years (*M* = 6.02, *SD* = 1.1). The children attended nursery and elementary schools in Warsaw.

*Study 4*. The participants in Study 4 were 226 individuals (171 women) aged 11–30 years (*M* = 13.10, *SD* = 6.04). They were students of elementary, middle, and high schools as well as university students from all over Poland.

*Study 5*. The participants in Study 5 were 741 individuals (425 women) aged 15–25 years (*M* = 18.30, *SD* = 3.04). They were students of middle and high schools as well as university students from all over Poland.

##### Measures and procedure

In all of the five studies, version A of TCIA was used. Apart from that, in each of those five studies we used different questionnaires and tests measuring characteristics directly related to creative imagination or creative abilities. In each study, the instruments were presented in a random order. The instruments used in particular studies are listed below.

*Study 1*. Perceived efficacy in using visual imagination was measured by the Vividness of Visual Imagery Questionnaire (VIVIQ) (Marks, 1973, 1995). The questionnaire consists of 32 items that are supposed to measure the degree to which the participant believes himself/herself to be capable of using imagination efficiently. An example item is: “In answering items 1 to 4, think of some relative or friend whom you frequently see (but who is not with you at present) and consider the picture that comes before your mind's eye. (1) The exact contour of face, head shoulders and body.” The reliability of the VIVIQ was high (α = 0.90).

*Study 2*. Creative imagination was measured using the Franck Drawing Completion Test (FDCT), successfully applied in earlier research on creativity (Dziedziewicz et al., 2013, 2014). FDCT is composed of 12 figures, placed in separate “windows.” The participants' task is to complete the initial figures in such a way that the end result takes the form of interesting drawings. There is no limit on the time taken to complete the task. The test is assessed on a three-point scale (0-1-2): no points are given for a conventional form, one point is given for a fairly complex form which partially stands out in its originality and unconventional approach, and two points are given for drawings with a rich, free, and unconventional form which are not strictly based on the initial symbol. The maximum score on the test is 24 points. The reliability of the FDCT was high (α = 0.83).

In the second study we also used a task that is a classic one in experiments concerning creative imagination and consists in drawing animals “from a different planet” (Generating Imaginary Animals; Ward, 1994). The participants were asked to list 20 animals that came to their mind (Listing Real Earth Animals). Next, they were to imagine a planet, completely different than Earth, on which a variety of plant and animal species existed. Based on the imagery generated, they made a detailed drawing of an imaginary creature as seen from the front and from the side, they gave it a name and named all the parts of its body. The images were assessed using an index applied in earlier studies (Ward, 1994; Ward and Sifonis, 1997; Ward et al., 2002)—the presence of untypical sense organs (creature attributes).

*Study 3*. In the third study, we used the Test of Creative Thinking-Drawing Production (TCT-DP) (Jellen and Urban, 1986). This test measures creative thinking defined in a broad way based on Urban's Components Model of Creativity (1996). The subjects are asked to complete an unfinished drawing. Detailed procedures of the TCT-DP are given in Urban (2004). Briefly, participants in this task are asked to complete an unfinished drawing that consists of a few shapes including a half-circle and a dot. Each participant is given a score of creative abilities based on 14 criteria: (1) continuations, (2) completions, (3) new elements, (4) connections made with a line, (5) connections made to produce a theme, (6) boundary breaking (fragment-dependent), (7) boundary breaking (fragment-independent), (8) perspective, (9) humor and affectivity, (10) manipulation of the material, (11) surreal or abstract drawings, (12) atypical combinations of figures and symbols, (13) non-stereotypical use of a certain element, and (14) speed. The final score given for the TCT-DP is a sum of points from all of these criteria. Previous studies (Gralewski and Karwowski, 2012; Karwowski and Gralewski, 2013) confirmed its value as a valid and reliable measure. In this study, the reliability of the TCT-DP was acceptable (α = 0.75).

*Study 4*. In Study 4, we used the verbal Alternate Uses Task inspired by Minnesota Tests of Creative Thinking (Torrance, 1962). The task was to come up with unusual uses for a can within a specified time (3 min). This task was scored in terms of fluency, flexibility, and originality of thinking.

*Study 5*. The circle test from the Torrance Tests of Creative Thinking (TTCT; Torrance, 1974) was used to measure divergent thinking (DT). The test consists of 20 empty circles arranged in 5 rows of 4 on the test sheet. The task is to create interesting drawings in them, trying to use all the circles within 10 min. The total number of circles used minus the number used for recurring themes gives an index of fluency (range: 0 to 20 points). This index is generally considered to be absolutely reliable because it relies on mechanical counting. Flexibility is indexed by the number of categories of themes considered; originality is indexed by the inverse of the frequency of occurrence of each concept in the whole sample (unique ideas score highest), and total originality score is the sum of the originality scores for each circle response generated by the participant (see Silvia et al., 2008; Plucker et al., 2011 for the advantages and limitations of different originality scoring methods).

The research program presented in this article was approved by the authors' university's Institutional Review Board. Written permission from the parents of the children participating was obtained prior to data collection. The participants were informed about the study and could withdraw at any time. All tests were scored by 3 research assistants (graduate students of psychology and education), trained in creativity tests scoring.

#### Results and discussion

The correlations between the three scales of TCIA and the dimensions of creative imagination and creative thinking are presented in Table 3. Additionally, in Table 4 we present the polychoric correlations between vividness, originality, and transformativeness of the TCIA and each of the 14 TCT-DP criteria.

**Table 3:** **Criterion validity analysis—Correlations of TCIA with VVIQ, FDCT, and creativity tests**.

	**Vividness**	**Originality**	**Transformativeness**
**Study 1 (*****N*** = **100)**			
VIVIQ	0.42^***^ [0.24, 0.57]	0.36^***^ [0.18, 0.52]	0.31^**^ [0.12, 0.48]
**Study 2 (*****N*** = **57)**			
Generating Imaginary Animals	0.02 [−0.24, 0.28]	0.45^***^ [0.21, 0.64]	0.32^*^ [0.06, 0.54]
FDCT	0.48^***^ [0.25, 0.66]	0.30^*^ [0.04, 0.52]	0.18 [−0.08, 0.42]
**Study 3 (*****N*** = **261)**			
TCT-DP	0.26^***^ [0.14, 0.37]	0.32^***^ [0.21, 0.42]	0.20^**^ [0.08, 0.31]
**Study 4 (*****N*** = **226)**			
Verbal fluency	0.13^*^ [0.00, 0.26]	0.26^***^ [0.13, 0.38]	0.13^*^ [0.00, 0.26]
Verbal flexibility	0.19^**^ [0.06, 0.31]	0.26^***^ [0.13, 0.38]	0.15^*^ [0.02, 0.28]
Verbal originality	0.14^*^ [0.01, 0.27]	0.26^***^ [0.13, 0.38]	0.13^*^ [0.00, 0.26]
**Study 5 (*****N*** = **741)**			
Figural fluency	0.14^***^ [0.07, 0.21]	0.05 [−0.02, 0.12]	0.07^^^ [0.00, 0.14]
Figural flexibility	0.14^***^ [0.07, 0.21]	−0.04 [−0.11, 0.03]	0.02 [−0.05, 0.09]
Figural originality	0.16^***^ [0.09, 0.23]	0.01 [−0.06, 0.08]	0.04 [−0.03, 0.11]

*95% confidence intervals are provided in brackets*.

^ p < 0.10;

* p < 0.05;

** p < 0.01;

*** *p < 0.001*.

**Table 4:** **Polychoric correlations between TCIA criteria and TCT-DP criteria**.

**TCT-DP Scoring Criteria**	**Vividness**	**Originality**	**Transformativeness**
Continuations (Cn)	0.12^*^	0.18^*^	0.08
Completions (Cm)	0.20^**^	0.27^***^	0.15^*^
New elements (Ne)	0.19^*^	0.28^^***^^	0.22^**^
Connections made with a line (Cl)	0.16^*^	0.12^*^	0.12^*^
Connections that contribute to a theme (Cth)	0.25^***^	0.27^***^	0.19^*^
Boundary breaking: fragment-dependent (Bfd)	0.09	0.14^*^	0.14^*^
Boundary breaking: fragment-independent (Bfi)	0.30^***^	0.11^*^	0.20^**^
Perspective (Pe)	0.38^***^	0.08	0.14^*^
Humor and affectivity (Hu)	0.26^***^	0.22^***^	0.10
Unconventionality: manipulation (Uca)	0.44^***^	0.12^*^	0.09
Unconventionality: surrealistic, abstract (Ucb)	0.14^*^	0.30^***^	0.07
Unconventionality: symbol-figure combination (Ucc)	0.21^**^	−0.04	0.13^*^
Unconventionality: symbols, signs (Ucd)	0.18^*^	0.26^***^	0.16^*^
Speed (Sp)	0.24^***^	0.19^*^	0.13^*^

* *p < 0.05*,

** p < 0.01;

*** *p < 0.001*.

In the case of measures pullback treated as referring directly to creative imagination (VIVIQ, FDCT, and Generating Imaginary Animals), seven out of nine correlation coefficients turned out to be statistically significant, with a generally substantial effect (median *r* = 0.32). Imagery abilities measured using VIVIQ turned out to correlate fairly consistently and with similar strength with all the three criteria—the most strongly with vividness (*r* = 0.42) and slightly less strongly with originality (*r* = 0.36) and transformativeness (*r* = 0.31). We obtained quite a similar picture of the relationship in the case of FDCT—the scores in this test were mainly linked with vividness (*r* = 0.48), less strongly with originality (*r* = 0.30), and the most weakly (as well as not significantly) with transformativeness (*r* = 0.18). By contrast, the number of untypical sense organs in the Generating Imaginary Animals task was independent of vividness (*r* = 0.02) but strongly related to the TCIA (*r* = 0.45) and transformativeness (*r* = 0.32).

In the case correlations between TCIA scales and measures of creative thinking, the situation was less clear. Only 11 out of 21 correlation coefficients were statistically significant, with a median of *r* = 0.12. The TCIA was related fairly consistently—though less strongly than with measures of imagination—to TCT-DP scores. Both vividness (*r* = 0.26) and originality (*r* = 0.32) as well as transformativeness (*r* = 0.20) were related to the overall score on this test. A more detailed analysis taking into account particular TCT-DP criteria (Table 4) unveiled more interesting patterns of relations. TCIA vividness was the most strongly related to TCT-DP unconventional manipulation (*r* = 0.44), perspective (*r* = 0.38), and fragment-independent boundary breaking (*r* = 0.30). Correlations between originality and TCT-DP criteria were weaker: they were the strongest in the case of using abstract elements (*r* = 0.30), introducing new elements into the drawing (*r* = 0.28), continuations of the existing elements (*r* = 0.27), and connections that contribute to a theme (*r* = 0.27). In the case of transformativeness, we found the strongest relations with new elements (*r* = 0.22) and boundary-breaking (fragment-independent) (*r* = 0.20).

Correlations between TCIA scales and the scores on tasks from Torrance's tests were both weaker and less systematic. What is interesting, the measures of creative imagination were almost completely unrelated to the classic scoring criteria of creative thinking tests (fluency, flexibility, originality) in the case of the figural test (only fluency was weakly related to vividness, *r* = 0.13). As regards the verbal test, the scores were the most consistently related to originality, which was related in an identical way (*r* = 0.26) to verbal fluency, flexibility, and originality. The relations between vividness and transformativeness and the measures of creative abilities were weaker, though significant (0.13 = *r* = 0.18).

The results of the first five studies confirm the validity of TCIA. Stronger relationships between the results obtained in the new test and established measures of creative imagination (VIVIQ, FDCT, Generating Imaginary Animals), compared to classic measures of creative abilities (also figural ones)^1^, support the statement that, measuring characteristics important for creativity, TCIA focuses to a greater extent on imagination rather than on the characteristics of thinking. Admittedly, the values of correlations between vividness, originality, and transformativeness and the measurements using other instruments developed for measuring imagination are not spectacularly high (the highest being *r* = 0.48 between FDCT and the vividness of imagination), but they are strong and consistent enough to be treated as confirming the criterion validity of the new measure. What is important, the obtained profile of various relations between the scales of TCIA and other measures also constitutes an argument supporting the validity of the new instrument. It is easy to notice that the attempts made so far to study creative imagination have focused only on its selected elements. For example, FDCT (Dziedziewicz et al., 2013) actually measures the vividness and, to a certain (smaller) extent, originality of creative imagination, but it does not measure transformativeness. The task of Generating Imaginary Animals (Ward, 1994; Ward and Sifonis, 1997; Ward et al., 2002) reveals much about originality and next to nothing about vividness. The new test makes it possible to systematically analyze all the three components important for the functioning of creative imagination without duplicating the measurement performed using any of the previous instruments and remaining relatively independent of creative thinking.

Assuming that the results presented in Studies 1–5 support the criterion validity of the new measure, the next important step was to determine its discriminant validity. For that purpose, we used measures of general intellectual ability (intelligence) and school achievement in different areas. Previous studies and meta-analyses (Kim, 2005; Karwowski and Gralewski, 2013) show that the relations between creativity and intelligence are not particularly strong (however, see Silvia, 2015, for an alternative position), and neither are the relations between creative abilities and school achievement (Gralewski and Karwowski, 2012; Gajda, in press; Gajda and Karwowski, Submitted). This is why we devoted Study 6 to checking the discriminant validity of the new test, correlating the results obtained in it with intelligence and school achievement.

### Discriminant validity (study 6)

#### Method

##### Participants

The participants in Study 6 were elementary school students. The sample was composed of 110 boys and 120 girls (total *N* = 230), whose mean age was 13.88 years (*SD* = 0.36). The participants were fifth-grade students from elementary schools across the whole Poland. The multilevel and multistrata sample selection made it representative for all Polish fifth-graders, with the exception of special school students and students from very small schools (below 10 students per grade). The sample was drawn from the registers of Polish Educational Information System (PEIS) (http://www.cie.men.gov.pl/index.php/sio.html). Four strata were distinguished according to school location (village, town below 20,000 inhabitants, city 20,000–100,000, city above 100,000) and school size. In each randomly chosen school, two classes were randomly invited to participate in the study.

##### Measures and procedure

Apart from the TCIA, all participants solved an intelligence test and school achievement test.

Intelligence. In order to measure intelligence, we used Raven's Progressive Matrices (RPM) (Raven et al., 2003). The reliability of RPM in this study was high (α = 0.85).

*Grade point average*. The grade point average for all school subjects from the semester preceding the research was used as a measure of school grades. The GPA was provided by students.

*School achievement*. As a measure of school achievement, we used the results of a school achievement test developed by the Educational Research Institute. This test measures three spheres of school achievement—math, reading, and overall language awareness. The test was developed and scaled according to item response theory (Rasch models is a one-parameter and graded partial credit model; Rasch, 1980) and has very good psychometrics properties—all items are well- fitted to the Rasch model (infit and outfit measures between 0.8 and 1.2). Moreover, the test information function at the average level of θ (a latent trait of the measured achievement) was high, and the standard error of measurement was low—translating into reliability between 0.86 and 0.88, depending on the scale (Jasińska and Modzelewski, 2012).

#### Results and discussion

Correlations between measures of intelligence and school achievement and the three scales of TCIA are presented in Table 5. As opposed to the relations with creative abilities, reported earlier, this time the profile of results is less clear. Vividness turned out to be a consistent correlate of intelligence (*r* = 0.29), GPA (*r* = 0.33), and achievement test scores in math (*r* = 0.28), reading (*r* = 0.24), and language awareness (*r* = 0.23). However, in the case of originality and transformativeness, the relations were less unambiguous and clearly weaker. Originality was significantly and positively, though weakly, related to school achievement in reading and language awareness, whereas transformativeness was related to GPA (*r* = 0.21) and competence in math (*r* = 0.20).

**Table 5:** **Discriminant validity analysis—correlations with intelligence and school achievement**.

**Study 6 (*N* = 230)**	**Vividness**	**Originality**	**Transformativeness**
IQ	0.29^***^	0.10	0.08
GPA	0.33^***^	0.09	0.21^**^
SAT Math	0.28^***^	0.05	0.20^***^
SAT Reading	0.24^***^	0.17^*^	0.09
SAT Language Awareness	0.23^***^	0.17^*^	0.11

* *p < 0.05*,

** p < 0.01;

*** *p < 0.001*.

The consistently positive relations found between intelligence, school achievement, and vividness suggest that their cause is not only vividness itself but the related ability to work persistently and thoroughly, closer to elaboration (Dziedziewicz and Karwowski, 2015). What may also be interesting is the role of transformativeness in learning math (probably especially geometry), which is confirmed by the relations found between skill in performing transformations in the imagination and achievement in math.

Study 6 brings 15 correlations, of which only nine are statistically significant, and the mean correlation coefficient (as well as median) obtained between intelligence and measures of imagination is *r* = 0.17. This result provides arguments in favor of the new test's discriminant validity.

Studies 1–5 make it justified to consider TCIA an instrument characterized by criterion validity, and Study 6 testifies to a good discriminant validity of the new test. The measurement of creative imagination using TCIA is quite consistently and strongly related to other measures of creative imagination, slightly less consistently and more weakly to creative ability tests, and the most weakly (as well as less systematically) to intelligence and school achievement. However, Studies 1–6 were based on the assumption that the three-factor structure of the test, assumed by the presented theoretical model, is reproduced in the data. In order to verify this assumption, in the next step we tested the construct validity of the new test, subjecting its results to confirmatory factor analysis as well as testing measurement invariance among men and women.

### Construct validity (studies 1–9 aggregated)

#### Method

##### Participants

The analysis covered data collected from 1740 people at different ages—the participants in Studies 1–9. In total, the sample consisted of 1200 women (69%) and 540 men (31%); 42 people did not give their gender. The participants' age ranged from 10 to 55 years (*M* = 16.33, *SD* = 4.72); most of them were students or university students taking part in various research projects using TCIA.

##### Measure and procedure

Sometimes the participants completed TCIA together with other tests, and sometimes it was the only test completed.

#### Results and discussion

**Figure 5:**
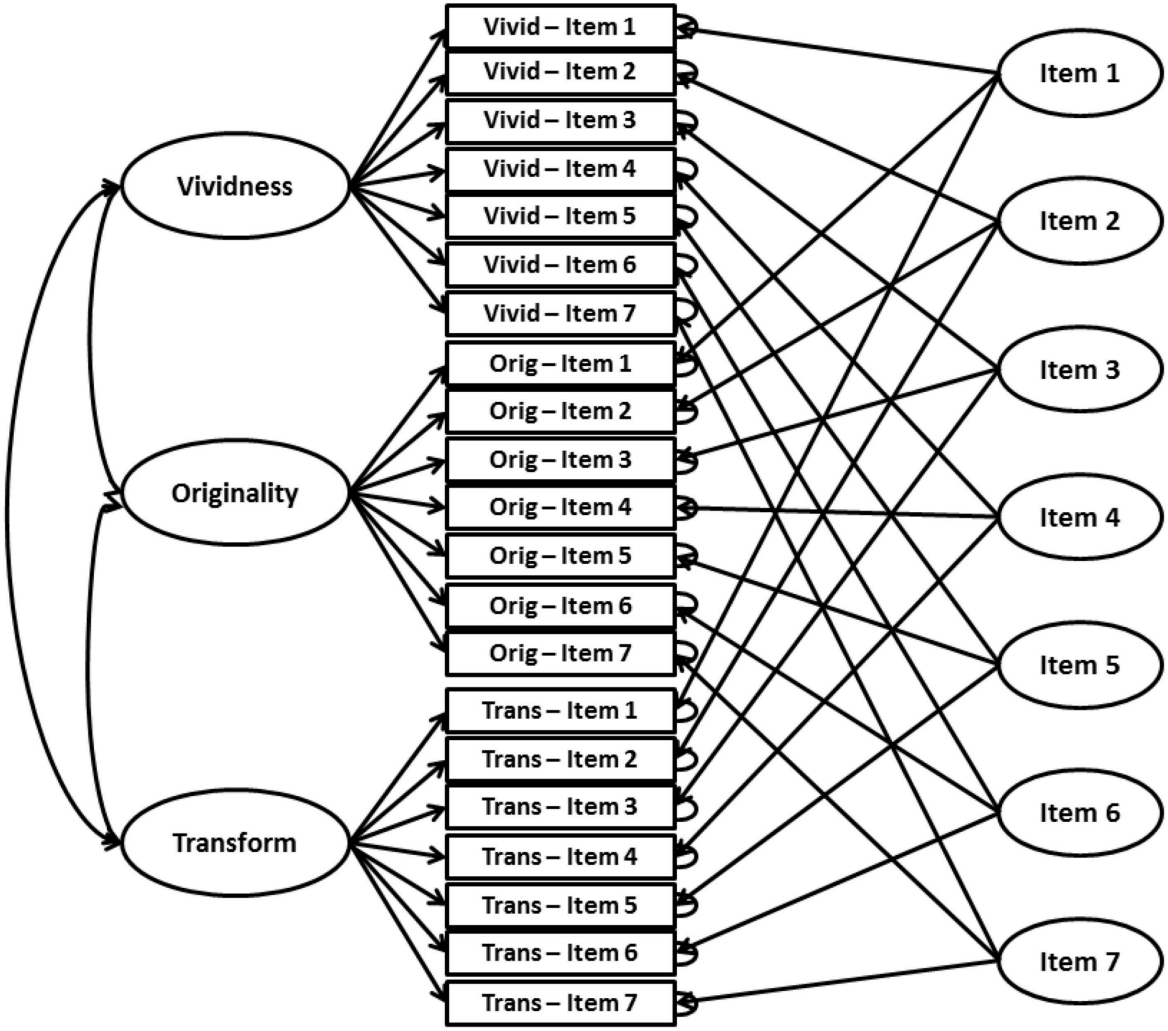
**Multi-trait, multi-method confirmatory factor analysis model testing for construct validity of the TCIA**.

In the first step, the data collected were subjected to confirmatory factor analysis in a design involving many traits and many methods. More specifically, we tested the fit of the three-factor model assumed on the basis of theory, while at the same time controlling the effect of the test's individual items (Figure 5).

The assumed theoretical model was confirmed (Table 6). Comparing the measures of fit with the commonly used criteria (Hu and Bentler, 1999; Kline, 2010), the values obtained should be considered acceptable.

**Table 6:** **CFA Model Fit Parameters**.

**Measures**	**Parameters**
χ^2^(*df*) / χ^2^/*df*	241.55 (165)/1.46
*CFI*/*TLI*	0.988/0.983
*RMSEA* (90% *CI*)	0.019 (0.013, 0.029)
**CORRELATIONS BETWEEN LATENT VARIABLES**	
Vividness-Originality	0.53^***^
Vividness-Transformativeness	0.39^***^
Originality-Transformativeness	0.56^***^
**FACTOR LOADINGS**	
Range of loadings on Vividness (mean)	0.60–0.67 (0.64)
Range of loadings on Originality (mean)	0.58–0.71 (0.65)
Range of loadings on Transformativeness (mean)	0.59–0.72 (0.68)
Items loadings (Vividness, Originality, Transformativeness)	
Item 1	0.62, 0.69, 0.70
Item 2	0.66, 0.71, 0.71
Item 3	0.65, 0.58, 0.68
Item 4	0.67, 0.66, 0.59
Item 5	0.65, 0.62, 0.67
Item 6	0.64, 0.59, 0.70
Item 7	0.60, 0.68, 0.71

*** *p < 0.001*.

The correlations between latent factors were moderately strong (0.39–0.56), and the factor loadings of the model estimated on the basis of polychoric correlations testify to a good validity of individual items (Hu and Bentler, 1999), considerably exceeding the literature-recommended minimum of 0.50. Thus, the construct validity of the model is confirmed by the obtained data.

#### Effects of gender and age on the TCIA results

The next step in analyses was to test TCIA measurement invariance according to gender. The fit of consecutive models with increasingly high constraint is presented in Table 7. The sample being large, we performed invariance assessment not on the basis of differences in the range of values of chi squared (sensitive to sample size), but by comparing the values of CFI and RMSEA between models. Following the recommendations found in the literature on the subject (Cheung and Rensvold, 2002; Chen, 2007), we consider a model to be invariant if CFI change between consecutive models does not exceed 0.01 and if the change in RMSEA does not exceed 0.02.

**Table 7:** **Analysis of test equivalence according to gender – invariance analysis (CFA)**.

**Model**	**χ^2^/*df***	**CFI**	**RMSEA (90% CI)**
Configural invariance	1.57	0.978	0.016 (0.014, 0.019)
Metric invariance	1.54	0.978	0.016 (0.013, 0.018)
Scalar invariance	1.71	0.968	0.018 (0.016, 0.021)

Even the most constrained model that tested scalar invariance had a very good fit, and differences in CFI between the models did not exceed 0.01, though comparing more and less constrained models does bring a decline in fit, slightly exceeding the critical values. However, given that the change in RMSEA between the least and the most constrained model is only 0.005, there are significant grounds to consider the models well-fitted and the test itself invariant according to gender.

The next step was to check the existence of gender differences in terms of the characteristics of creative imagination. For this purpose, three latent variables: vividness, originality, and transformativeness were predicted by gender. The model was well fitted to data (χ^2^/*df* = 1.42, *CFI* = 0.988, *RMSEA* = 0.018), and the effect of gender in all three cases turned out to be statistically significant. More specifically, women exhibited a higher level of vividness (β = 0.25, *p* < 0.001), originality (β = 0.19; *p* < 0.001), and transformativeness (β = 0.17, *p* < 0.001).

An analogous model with age as a predictor was also well fitted (χ^2^/*df* = 2.36, *CFI* = 0.959, *RMSEA* = 0.032); age was a statistically significant positive predictor of vividness (β = 0.19, *p* < 0.001), originality (β = 0.14, *p* < 0.001), and transformativeness (β = 0.078, *p* < 0.01).

The analyses presented above confirm the construct validity of TCIA. As assumed, the test has a three factor structure, and the three components of creative imagery are significantly and moderately correlated. At the same time, however, correlations between them are not strong enough to make them indistinguishable from one another. Individual items load on the latent variables strongly enough to justify the conclusion about their criterion validity. These data testify to the good validity of the measure.

We devoted the next three studies (7–9) to assessing the reliability of TCIA. Study 7 concerned testing the consistency between the judges scoring TCIA based on detailed guidelines provided in the manual (Jankowska and Karwowski, 2015). Studies 8 and 9 concerned test-retest reliability. The whole research concludes with a presentation concerning reliability assessed as the test's internal consistency.

### Interjudge reliability (study 7)

#### Method

##### Participants

The participants were four judges (all female, mean age *M* = 26 years) trained in TCIA scoring.

##### Measures and procedure

All the judges took part in a training devoted to details of TCIA scoring and acquainted themselves with the test manual (Jankowska and Karwowski, 2015). Next, each of them was asked to score 100 test sheets.

#### Results and discussion

For each of the three TCIA scoring criteria, we computed intercorrelations between the judges' ratings as well as their consistency using Cronbach's α and the intraclass correlation coefficient (ICC) (Table 8).

**Table 8:** **The reliability of judges scoring 100 randomly selected images generated in TCIA**.

**Study 7 (*N* = 100 drawings)**	**Judge 1**	**Judge 2**	**Judge 3**	**Judge 4**
**Vividness (α** = 0.91**, ICC** = **0.89)**				
Judge 1	1			
Judge 2	0.78	1		
Judge 3	0.82	0.76	1	
Judge 4	0.64	0.60	0.67	1
**Originality (α** = 0.90**, ICC** = **0.89)**				
Judge 1	1			
Judge 2	0.74	1		
Judge 3	0.61	0.67	1	
Judge 4	0.75	0.76	0.69	1
**Transformativeness (α** = 0.92**, ICC** = **0.91)**				
Judge 1	1			
Judge 2	0.84	1		
Judge 3	0.88	0.84	1	
Judge 4	0.70	0.53	0.68	1

*All correlations are statistically significant (p < 0.001)*.

In all situations, interjudge consistency was very high and comparable between the criteria. In all cases, α was equal to or higher than 0.90 (originality α = 0.90, vividness α = 0.91, and transformativeness α = 0.92), with slightly lower but still acceptable ICC values (vividness and originality ICC = 0.89, transformativeness ICC = 0.91).

The fact that briefly trained judges equipped with example assessments of TCIA products are capable of scoring the products of this test very similarly testifies to its good reliability. High consistency is a precondition of precise measurement. It is worth noting that the values we obtained are similar to those usually obtained in the case of other creativity tests, for example TCT-DP (Kālis et al., 2014) or TTCT (Dziedziewicz et al., 2013). This makes it legitimate to believe that even though TCIA scoring is a multifaceted and seemingly complex and difficult process, following our recommendations and using the examples provided does in fact make it possible to obtain highly reliable data. In the next two studies, we tested the reliability of TCIA in time: in Study 8 we used the same version of the test twice, whereas in Study 9 we used version B. In the final step, using aggregated data from all the studies described in this paper, we present data on the internal consistency of TCIA.

### Test–retest reliability (studies 8–9)

#### Method

##### Participants

*Study 8*. The participants in Study 8 were 86 people (43 women) aged 13 to 15 years (*M* = 14.02, *SD* = 0.84). They were high-school students from a large city in central Poland.

*Study 9*. The participants in Study 8 were 39 people (29 women) aged 13 to 14 years (*M* = 13.75, *SD* = 0.47). They were middle-school students from a big city in central Poland.

##### Measures and procedure

In Study 8, TCIA version A was used twice with a 3-week interval. In Study 9, there were 5 weeks between the measurement sessions using versions A and B of TCIA.

#### Results and discussion

Test-retest correlations between measurement using the same version of the test with an interval of 3 weeks were very high (*r* = 0.89 for vividness, *r* = 0.91 for originality, and *r* = 0.98 for transformativeness, all *p*'s < 0.001), testifying to very high measurement reliability (Table 9).

**Table 9:** **Test–retest reliability and internal consistency of TCIA**.

	**Vividness**	**Originality**	**Transformativeness**
Study 8 (test–retest, 3 weeks) *N* = 86	0.89^***^	0.91^***^	0.98^***^
Study 9 (A-B, 5 weeks), *N* = 39	0.63^***^	0.55^***^	0.43^***^
**Studies 1–9 (internal consistency)**			
Cronbach's α	0.83	0.84	0.86
H (CFA)	0.83	0.84	0.87

*** *p < 0.001*.

In the case of studies using versions A and B of the test, with an interval of 5 weeks between measurements, correlations were still fairly high—they ranged from *r* = 0.43 for transformativeness, through *r* = 0.55 for originality, and *r* = 0.63 for vividness (all *p*'s < 0.001).

The high values of test-retest correlations, especially those from Study 8, combined with the high interjudge consistency presented earlier, testify to the good reliability of TCIA measurement. The final step of our analyses was to test the internal consistency of each scale of TCIA. For this purpose, we used aggregated data from all the studies presented in this paper.

### Internal consistency (studies 1–9 aggregated)

#### Method

##### Participants

The analysis covered data collected from 1740 people at different ages—the participants in Studies 1–9. In total, the sample consisted of 1200 women (69%) and 540 men (31%); 42 people did not give their gender. The participants' age ranged from 10 to 55 years (*M* = 16.33, *SD* = 4.72); most of them were students or university students taking part in various research projects using TCIA.

##### Measures and procedure

All the participants solved TCIA, sometimes together with other tests and self-report measures and sometimes as the only test.

#### Results and discussion

We assessed internal consistency using the values of Cronbach's α and the *H* coefficient—composite reliability specific to confirmatory factor analysis (Hancock and Mueller, 2001). The scale on which the criteria were measured being short (0-1-2 in the case of each criterion and each individual item), we computed internal consistency on the basis of the matrix of polychoric correlations estimated in Mplus 7.1 (Muthén and Muthén, 2015).

The two methods yield very similar estimations of internal consistency. In the case of vividness and originality, the internal consistency indices have very similar values (0.83 for vividness and 0.84 for originality), whereas in the case of transformativeness internal consistency is α = 0.86 and *H* = 0.87.

These values demonstrate the good reliability of the test, especially as both coefficients applied depend on the number of items in a scale, and each scale of TCIA consists of a relatively small number of items (7). Internal consistency exceeding 0.80 may be regarded as highly acceptable and testifying to the good quality of TCIA measurement.

## General discussion

Creative functioning requires different abilities that very likely also include visual creative imagination. According to the conjunctional model of creative imaging ability (Dziedziewicz and Karwowski, 2015), the key abilities are those of visualizing, transforming, and enriching imagery, as well as combining them into new wholes. It must be stressed that this is not only the domain of children with vivid imagination or artists, but the quality of every person's mind, which facilitates visualizing problems and looking at them in new ways, leading to original solutions being generated more easily. This is what makes it so important to have valid and reliable tests of creative imagination. The existing instruments for measuring visual creative imagination have many shortcomings; for example, they have unclear theoretical roots, copy the scoring standards of divergent thinking tests, or measure only selected elements of imagery abilities, mainly vividness and originality. The detailed analysis of problems connected with measuring creative imagination, described in this paper, constituted the basis for the assumptions adopted in the construction of TCIA.

The aim of the presented research was to document the quality of measurement using TCIA. Four issues must be stressed in this conclusion. First, the results of correlational studies using other measures of creative imagination and creative thinking confirm the criterion validity of the test (Studies 1–5). Second, the study of creative imagination using TCIA combined with the measurement of intelligence and school achievement provided sufficient evidence for the discriminant validity of the new instrument (Study 6). Third, aggregated data from all studies subjected to confirmatory factor analysis provided arguments in favor of the test's construct validity—its three-factor structure was confirmed. Finally, both versions of the test as a whole are reliable, and this also applies to each of their scales (Studies 7–9).

We have demonstrated the measurement invariance of TCIA in case of gender. It allowed us to test for gender differences in the latent means of TCIA scales. Although the differences were small in terms of the effect size, females outperformed males in vividness, originality and transformativeness. Similarly, there was small, but positive effect of age, with older participants achieving higher results in the TCIA. Gender differences obtained in our studies fit well with previous studies and show that not only women usually obtain higher scores than men in self-assessed imaginative abilities (mainly vividness) (Harshman and Paivio, 1987; Narchal and Broota, 1988), but they also do in terms of imaginative abilities (Karwowski, 2009; Lau and Cheung, 2010). These differences may be due to girls' engaging more in role-playing or personal fantasy plays than boys during preschool years (Werebe and Baudonniere, 1991). Furthermore, girls around 4 to 5 years of age have been observed to engage in role-playing and in personal play fantasy twice as often as the boys of a similar age group (Jones and Glenn, 1991). One of the most widely replicated findings in the research on imaginary companions is that girls are more likely to have them than boys (Singer and Singer, 1992; Carlson and Taylor, 2005).

Summing up, it should be said that TCIA is characterized by high validity and reliability in measuring visual creative imagination. Moreover, several findings presented in this paper may be interesting not only as confirmations of the quality of the test. The generally weak association between creative imagination and divergent thinking or intelligence we have obtained replicates previous findings that generally show low correlations between imagination and creativity (Schmeidler, 1965). Although generally those correlations are statistically significant and positive, they rarely exceed the value of *r* = 0.30, hence providing good arguments that these constructs are relatively independent aspects of creative abilities (see e.g., Rhodes, 1981; Russ and Grossman-McKee, 1990; Dziedziewicz et al., 2013). Usually, correlations between divergent thinking and vividness of imagery are higher than those with transformativeness (LeBoutillier and Marks, 2003). Similarly, usually creative imagination is more strongly related to originality than to fluency of thinking (Dziedziewicz et al., 2013, 2014).

### Limitations and future directions

The research presented here had a correlational character. Experimental research would make it possible to check, in a controlled way, whether the complexity of different imagery transformations was reflected in the Transformativeness scale. Further research should capture the dynamics of the process of image transformation, as has been done in the analysis of reaching solutions in creativity tests (Beaty et al., 2014). Perhaps it is even worth attempting to combine the testing of creative imagination with neuropsychological methods such as EEG or MRI (Fink and Benedek, 2012).

What seems very promising is the profile-based approach in the measurement of creative imagination, which shows the complex and multifaceted nature of this disposition. In the future, using the experience gathered when classifying the profiles of other multiscale tests and questionnaires, it is worth developing an objective and reliable system of defining profiles of creative imagery abilities by means of statistical procedures. Its usefulness for scientific purposes, but above all in individual assessment and in choosing the type of stimulatory interventions, will be invaluable.

The results presented in this paper focused especially on the version of TCIA that is intended for group research. Another paper devoted to a version developed for individual studies that includes the study of children aged 4 and older is in preparation.

At present, plans also exist to perform a cultural adaptation of TCIA in order for the instrument to be successfully used in other countries (outside Poland), in research on imagination—its nature, development, and determinants, in comparative cross-cultural studies.

## Conclusion

The results of our studies to date on the validity and reliability of the TCIA make it legitimate to say that TCIA is a measure with good—or even very good—psychometric properties and a clear theoretical basis.

What makes it valuable is, above all, the emphasis it gives to the complexity and multidimensionality of visual creative imagination, in which it stands out favorably against other tests measuring this disposition. This test enables a systematic analysis of all the three components important to the functioning of creative imagination while remaining relatively independent of creative thinking. Due to the possible application of the instrument in assessment and intervention practice—in measuring the effectiveness of stimulatory interventions—the fact that that TCIA exists in two versions is also of significance.

### Conflict of interest statement

The authors declare that the research was conducted in the absence of any commercial or financial relationships that could be construed as a potential conflict of interest.

## Footnotes

^1^Table 3 presents 95% confidence intervals around Pearson's *r*s, allowing for direct comparisons of different correlations. However, to provide a more synthetic comparison of correlation coefficients obtained between TCIA scales and other tests, we followed a two-step procedure. First, using a multilevel meta-analysis (Cheung, 2014; Karwowski and Lebuda, 2015), we calculated the correlations between TCIA scales and criterion measures (VIVIQ, Generating Imaginary Animals, FDCT). Then, we provided a similar meta-analysis for correlations between the TCIA scales and other creativity measures. The meta-analytically obtained correlation between TCIA and creative imagination measures was estimated at *r* = 0.34 (95% *CI*: 0.27,0.41), while the correlation between TCIA and other creativity measures was at *r* = 0.135 (95% *CI*: 0.038,0.23). Second, as confidence intervals across *r*s do not overlap, we conclude that these coefficients differ significantly from each other.
